# A Scalable Microfluidic Platform for Bacterial Cellulose Production and Characterization

**DOI:** 10.1021/acsomega.5c04233

**Published:** 2025-11-20

**Authors:** Brunella Corrado, Francesca Mauro, Vincenza De Gregorio, Elena Lagreca, Concetta Di Natale, Raffaele Vecchione, Paolo Antonio Netti

**Affiliations:** † Interdisciplinary Research Centre on Biomaterials, 9307 University of Naples Federico II, Piazzale Tecchio 80, 80125 Naples, Italy; ‡ Istituto Italiano di Tecnologia, 378791 Center for Advanced Biomaterials for Health Care CABHC@CRIB, Largo Barsanti e Matteucci 53, 80125 Naples, Italy; § Department of Biology, Complesso Universitario Di Monte S. Angelo, 9307 University of Naples Federico II, Via Cinthia, 80126 Naples, Italy; ∥ Department of Chemical, Materials and Industrial Production Engineering (DICMaPI), Piazzale Tecchio 80, 80125 Naples, Italy; ⊥ Institute of Applied Sciences and Intelligent Systems (ISASI), National Research Council of Italy (CNR), Via Campi Flegrei 34, Pozzuoli, 80078 Naples, Italy

## Abstract

Bacterial cellulose (BC) is an attractive biomaterial due to its biocompatibility, mechanical strength, and tunable properties. However, there is a need for a rapid and cost-effective platform to screen BC synthesis processes and optimize culture conditions. This study focuses on the bacterium *Komagataeibacter xylinus* as a model system to explore BC production. Existing methods for studying BC synthesis often rely on traditional large bioreactors, which are resource-intensive and lack the precision needed for the high-throughput testing of culture parameters. Here, we propose a scalable microfluidic platform that enables real-time monitoring of cellulose fibril secretion via confocal microscopy, facilitating quantitative characterization of cellulose network properties. To validate the platform, we tested variable concentrations of yeast extract and glucose, two essential culture parameters known to affect BC production, using a gradient-mixing chamber integrated into the device. Structural analyses performed using both confocal and scanning electron microscopy (SEM) revealed significant correlations between the nutrient concentration and BC ultrastructure. Specifically, we found that higher yeast extract concentrations result in denser and more compact matrices, whereas increased glucose levels produce more porous structures.

## Graphical abstract

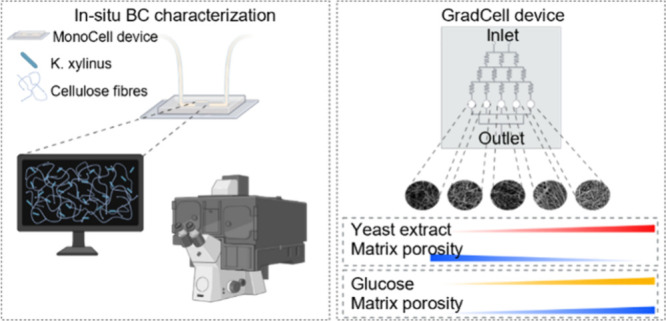

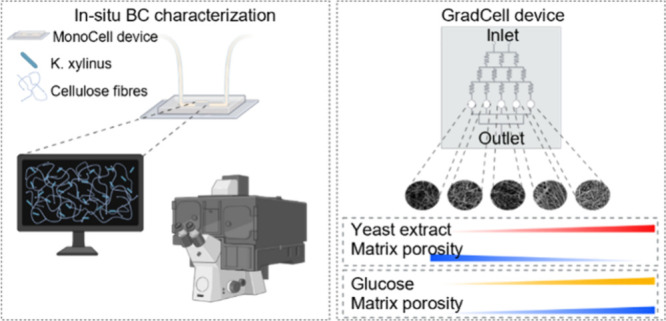

## 1 Introduction

Cellulose, a natural macromolecule composed of glucose molecules linked by glycosidic bonds, is conventionally synthesized by plants as vegetable cellulose, serving important roles in the textile and food industries.^1^ Alternatively, certain bacterial strains synthesize this polymer as a protective polymeric biofilm in a pellicle-like form.^2^ Among these strains, acetic acid bacteria like *Komagataeibacter xylinus* (*K. xylinus*) are prolific producers,^3,4^ synthesizing nanoscale fibers that form robust, elastic three-dimensional networks. Bacterial cellulose (BC) exhibits distinct properties from its plant counterpart, including high water absorption, moldability, biocompatibility, elasticity, and chemical purity.^5,6^ Remarkably, BC’s production avoids the complex chemical processes required to remove plant cellulose contaminants like lignin and hemicellulose, aiding production under controlled conditions to yield highly pure and customizable biopolymers.^7^ These features have propelled BC into the forefront of research in the cosmetic and biomedical fields, where its purity and biocompatibility enable applications ranging from wound dressing^8^ to drug delivery systems.^9^ Traditional methods for BC production typically rely on large bioreactors operating under static or agitated conditions. Despite their widespread use, these procedures often face several limitations.^10^ In particular, these systems lack the capability for real-time monitoring of process parameters and offer limited control over biofilm formation and its modification, making it challenging to optimize production conditions. Additionally, the high consumption of culture media and the inability to facilitate high-throughput testing significantly increase production costs, particularly in large-scale systems. Nevertheless, some researchers have focused on real-time monitoring of the cellulose production. For instance, Opitz et al. developed and evaluated in situ microscopy (ISM) as a noninvasive method for real-time monitoring of enzymatic cellulose hydrolysis.^11^ ISM enables real-time monitoring of particle size and reaction progress in bioreactors without sample withdrawal. However, its complexity, need for advanced calibration, and susceptibility to turbidity and maintenance challenges make it more suitable for large-scale systems. In this context, microfluidic technologies have emerged as powerful tools for real-time monitoring in bioprocesses, offering unparalleled precision and control over experimental conditions.^12^ These systems enable the integration of dynamic monitoring capabilities, allowing researchers to track biofilm formation under dynamic conditions,^13–15^ nutrient gradients, and cellular responses in real time.^16,17^ Moreover, by reducing reagent volumes and costs while facilitating high-throughput testing, microfluidic platforms provide a scalable and efficient alternative to traditional methods.

Most of the studies using microfluidics focus on bacterial biofilm formation under shear stress in microfluidic devices designed to mimic pathological conditions. These systems often utilize PDMS bonded on glass, which inherently induces shear stress during biofilm formation.^18–20^ Additionally, another study^21^ explores microfluidics for cellulose production monitoring, but focuses on cellulose dispersions rather than true biofilms, preventing a proper investigation of biofilm dynamics. In summary, no continuous bacterial cellulose biofilms have been reported in microfluidic systems so far. Existing microfluidic platforms for BC production rely on the application of shear stress and often include physical constraints, such as pillars, to guide cellulose deposition.^22^ These approaches, however, lead to a discontinuous and spatially confined BC network. In contrast, our system enables the formation of a continuous and homogeneous bacterial cellulose layer without the need for structural confinement.

However, to replicate physiological batch production conditions, it is essential to design configurations that eliminate direct shear stress. To this end, we introduce a novel double-compartment microfluidic chip designed for the fermentation of *K. xylinus* and subsequent production of cellulose biofilm. This device not only enables the real-time confocal microscopy monitoring of cellulose fibril secretion but also facilitates quantitative analyses of the newly synthesized cellulose fibrils’ fraction and intersections, avoiding shear stress and enabling efficient real-time monitoring of cellulose biofilm formation while reliably reproducing static batch conditions. Moreover, the proposed single-chamber microfluidic device has been integrated into a more complex configuration to provide concentration gradients, allowing us to explore the influence of various growth culture conditions on the characteristics and formation of cellulose biofilm, thereby broadening the scope of microfluidic applications in biopolymer research. Hereafter, we refer to the single-chamber device as MonoCell (MC) and the gradient-mixing chip as GradCell (GC).

## 2 Materials and Methods

### 2.1 Bacterial Strains and Growth Conditions

*Komagataeibacter xylinus* (ATCC 53524) was purchased from the American Type Culture Collection and used for all the experiments involving BC production. All chemicals required for culturing *K. xylinus*, including glucose, peptone, yeast extract, Na_2_HPO_4,_ and citric acid, were purchased from Merck.

*K. xylinus* was maintained in the HS liquid medium described by Hestrin and Schramm,^23^ which contained 2.0% glucose (w/v), 0.5% yeast extract (w/v), 0.5% peptone, 0.25% Na_2_HPO_4_ (w/v), and 0.15% citric acid (v/v) in 1 L of distilled water. The pH was adjusted by adding 12 M HCl. Before inoculation, the culture medium was autoclaved at 121 °C for 20 min, while temperature-sensitive solutions, such as glucose, were sterilized using a 0.2 μm filter. A suspension of *K. xylinus* was added to 15 mL of HS medium in a 50 mL Falcon tube, achieving a concentration of 1 × 10^6^ bacteria per mL. To ensure a reproducible bacterial concentration at the start of each experiment, a fresh culture was inoculated from a mother culture and incubated for 72 h at 30 °C. This incubation period allowed for a consistent growth over time in the liquid medium. Before the desired volume was withdrawn for subsequent inoculation, the working culture was vortexed for 15 s to ensure homogeneity of the solution.

### 2.2 Device Fabrication

#### 2.2.1 MonoCell Platform

The MonoCell (MC) platform was developed using replica molding of polydimethylsiloxane (PDMS; Sylgard 184; Mascherpa), with a 1 mm high poly­(methyl methacrylate) (PMMA) slab as the master mold. The PMMA master mold was designed in AutoCAD (Autodesk, USA) and carved by using a micromilling machine (Minithech CNC Mini-Mill). The PMMA mold was then used to replicate the design in PDMS, with a polymer-to-curing agent ratio of 10:1 (w/w).

The MC device comprises a double-layer PDMS chip with a 0.2 μm polycarbonate (PC) membrane sandwiched between the two layers. The lower PDMS layer acts as the culture chamber, comprising a central microchannel (1.2 mm wide × 50 mm long × 0.6 mm high) and a central circular chamber (12 mm diameter × 0.6 mm high), which allows for the visualization of the cellulose synthesis process.

The upper PDMS layer serves as the flow chamber, mirroring the geometry of the lower layer but with a shorter microchannel length (1.2 mm wide × 45 mm long × 0.6 mm high). This design ensures that the inlet and outlet of both layers do not overlap, enabling easy loading of the bacterial suspension into the lower layer.

The embedded PC membrane serves as a barrier for the bacterial suspension, preventing it from being carried away by the overlying tangential flow and acting as a gas diffusion barrier to efficiently control oxygen exchange.

The PDMS prepolymer was poured onto each PMMA mold, degassed to remove air bubbles, and then incubated for 60 min at 80 °C until fully cured. Once cured, the PDMS was detached from the mold and trimmed with a scalpel at the edges. Inlets and outlets for both layers were produced using a 1.5 mm biopsy punch, and the central chamber of the bottom layer was punched using a 12 mm hole punch. The PDMS molds for both layers were then assembled by using oxygen plasma treatment to promote adhesion. First, the lower layer was etched onto a glass microscope slide (24 mm wide × 60 mm long) via plasma treatment in oxygen (1 min at 50 W). Subsequently, a squared sample of the PC membrane (15 mm length) was placed between the two layers over the culture chamber. The membrane was irreversibly bonded to the lower PDMS layer using a 5% Aminopropyltriethoxysilane (APTES) solution, a common amino silane for surface silanization and functionalization, as described by Aran et al.^55^

Briefly, a commercial APTES solution was diluted to 5% in water and heated at 80 °C on a hot plate for 20 min. The PC porous membrane and the lower PDMS layer were oxygen-treated for 1 min at 50 W. After oxygen activation, the PC membrane was placed on the PDMS, and the APTES solution was dropped on the PC membrane and incubated at 80 °C for 5 min. To bond the upper PDMS layer in a sandwich structure, both the upper PDMS layer and the bonded laminate were oxygen-activated, brought into contact, and pressed together. The entire setup was incubated at 80 °C overnight to achieve irreversible bonding of the two PDMS layers.

Before bacterial culture, the MonoCell device, tubes, and connectors were sterilized by autoclaving at 121 °C for 20 min. A peristaltic pump (Cole Parmer) was used to set a flow rate of 200 μL/min to deliver appropriate nutrients to the bacterial culture. For the microfluidic culture, a suspension of *K. xylinus* at a concentration of 1 × 10^6^ bacteria per mL was introduced into the lower layer’s channel via pipetting. Once the suspension had filled the observation chamber, microcaps with a diameter of 1.5 mm were used to seal the inlets and outlets. Tubes and connectors were then attached to the inlets and outlets of the upper layer, linking them to a peristaltic pump. The whole setup was placed in a humidified incubator at 30 °C to support the BC culture process. The design of the assembled device enables the continuous monitoring of cellulose layer growth and development via optical microscopy.

Simultaneously, a control culture was established in a multiwell plate to aid a comparative analysis of the cellulose layers produced under both dynamic and static conditions. After 72 h of culture, the cellulose layers from both setups were harvested and assessed for differences in matrix composition, degree of polymerization, and characteristics of the cellulose fibrils.

#### 2.2.2 Gradient Mixing Chip

The GradCell (GC) mixing platform was fabricated using replica molding of polydimethylsiloxane (PDMS; Sylgard 184; Mascherpa) with a 1 mm high poly­(methyl methacrylate) (PMMA) slab serving as the master mold. The PMMA master mold was designed in AutoCAD and fabricated using a micromilling machine (Minitech CNC Mini-Mill). This PMMA mold was then employed to replicate the design in PDMS, using a polymer-to-curing agent ratio of 10:1 (w/w).

This gradient mixing chip was employed to assess the effects of varying concentrations of either yeast extract or glucose on the bacterial culture and cellulose matrix production. The GC device comprises a double-layer PDMS chip, with a 0.22 μm polycarbonate membrane interposed between the two layers and irreversibly bonded as described in the previous section. The top layer features two inlets leading to a network of serpentine microchannels (300 μm wide × 300 μm high), which branch out into five circular culture chambers (4 mm diameter × 300 μm high), all ultimately converging into a single outlet. The bottom layer consists of five additional culture chambers, each 4 mm in diameter, connected to the inlets and outlet by rectangular channels of the same dimensions. The inlets, outlets, and culture chambers were precisely fabricated using biopsy punches of appropriate diameters (1.5 mm for inlets and outlets and 4 mm for culture chambers).

To estimate the concentration of solutions in each culture chamber downstream of the serpentine channels, the following equation was used, based on a previously reported formula:^24^

$$C\left(i,N\right)=\frac{\left(N-i\right)C_{1}+iC_{2}}{N}$$

where *C*(*i*, *N*) represents the concentration in the culture chambers (at the end of the sinusoid channels), *N* is the number of branches in the microfluidic network upstream of the chambers, and *i* is the index of each concentration. In our setup, *N* equals 4, as the chambers are four steps from the inlets, with *i* ranging from 0 to 4, corresponding to the five culture chambers at the outlet. By variation of the initial concentrations of *C*_1_ and *C*_2_, different final concentrations were achieved in the chambers. As the solutions passed through the gradient generator, they mixed and diluted repeatedly, creating a stable concentration gradient at the outlets.

For each experiment, a suspension of *K. xylinus* was introduced into the culture chambers of GC, and two culture media were pumped into the system through the corresponding inlets. One inlet received the medium with the highest concentration of the supplement of interest (either yeast extract or glucose), while the other inlet received the medium with the lowest concentration (0%). To start the culture, the entire setup was connected to a peristaltic pump set at 50 μL/min to establish laminar flow and then placed in a humidified incubator at 30 °C with 80% humidity.

In the first gradient mixing experiment, a medium containing 0.5% yeast extract was introduced through inlet 1, while a medium with 0% yeast extract was introduced through inlet 2. The gradient setup produced the following final concentrations of yeast extract at the five outlets: 0, 0.125, 0.25, 0.375, and 0.5% (w/v). In the other gradient mixing experiment, the medium at inlet 1 contained 7% glucose, while inlet 2 received a medium with 0% glucose. This setup provided the following final concentrations of glucose at the five outlets: 0, 1.75, 2.5, 5.25, and 7% (w/v).

After 72 h of culture, cellulose layers obtained from both experiments were collected for further analysis.

### 2.3 Ultrastructural Analysis

Cellulose layers harvested from the control culture, single chamber chip, and gradient chamber chip were first fixed using 4% paraformaldehyde, followed by a secondary fixation with 2.5% glutaraldehyde in 0.1 M sodium cacodylate buffer for 2 h at room temperature. The samples were then washed twice with 0.1 M cacodylate buffer at pH 7.2, each wash lasting 10 min. An overnight postfixation was performed at 4 °C using 1% osmium tetroxide, buffered in 100 mM cacodylate, pH 7.2. Then, the samples underwent a graded ethanol dehydration series before being critically point-dried with liquid carbon dioxide using a Critical Point Dryer (EM CPD300). Samples were mounted onto metal stubs and sputter-coated with gold–palladium at 10 mA. They were then analyzed under a Leica S400 scanning electron microscope (SEM). High-resolution images (1024 × 768 pixels) were captured at magnifications up to 50,000× and analyzed using ImageJ software for initial segmentation. The “DiameterJ” plugin^56^ was subsequently used to quantify the diameter of cellulose bundles and fibers. Additionally, the DiameterJ plug-in was used to extract measurements of the porosity percentage, mean pore area, and intersection density to characterize the overall bacterial cellulose network. All of these parameters were provided directly from the plugin. In detail, the porosity percentage was calculated as the ratio of black pixels to the total number of pixels in the image, according to eq 1 thus quantifying the structural integrity and porosity of the cellulose layers:

$$\mathrm{porosity}=\frac{\mathrm{Total}\;\mathrm{Number}\;\mathrm{of}\;\mathrm{Black}\;\mathrm{Pixels}}{\mathrm{Total}\;\mathrm{Number}\;\mathrm{of}\;\mathrm{Pixels}\;\mathrm{in}\;\mathrm{Image}}$$ 1

The mean pore area was obtained as the ratio of the total number of black pixels measured in the pores to the total number of pores in the image and then converted into length units (μm^2^), as reported in eq 2:

$$\mathrm{Mean}\;\mathrm{pore}\;\mathrm{area}=\frac{\mathrm{Total}\;\mathrm{Number}\;\mathrm{of}\;\mathrm{Black}\;\mathrm{Pixels}\;\mathrm{in}\;\mathrm{Pores}}{\mathrm{Total}\;\mathrm{Number}\;\mathrm{of}\;\mathrm{Pores}}$$ 2

Finally, fiber intersection density was evaluated as the ratio of the number of fiber overlaps to the total number of pixels in the image and then converted into a length unit (ints/μm^2^), as reported in eq 3:

$$\mathrm{Inter}\sec\mathrm{tion}\;\mathrm{density}=\frac{\mathrm{Number}\;\mathrm{of}\;\mathrm{Fibre}\;\mathrm{Overlaps}}{\mathrm{Total}\;\mathrm{Number}\;\mathrm{of}\;\mathrm{Pixels}\;\mathrm{in}\;\mathrm{Image}}$$ 3

### 2.4 Confocal Image Analysis

To enable real-time monitoring of cellulose secretion in the MonoCell, a 1 mL suspension of *K. xylinus* mixed with 0.02% Calcofluor white was injected into the lower channel of the device, filling the observation chamber. The system, including tubes and connectors to the peristaltic pump, was housed in a microscope stage top incubator with controlled conditions (temperature = 30 °C, humidity >80%). An 18 h time-lapse was conducted to capture early cellulose fibril secretion. Confocal microscopy at 6, 12, 18, 24, 48, and 72 h postinoculation provided images for quantitative analysis using ImageJ software, focusing on cellulose fraction (CF) and the number of intersections. In detail, after converting the image into binary form, CF was defined as the ratio of clear pixels to the total number of pixels, according to eq 4):

$$\mathrm{CF}=\frac{N_{C}}{N_{C}+N_{B}}$$ 4

where *N*_C_ and *N*_B_ represent the number of bright (cellulose) and dark (noncellulose) pixels, respectively, within a selected region of interest (ROI).

The number of intersections was evaluated by using the “Skeleton Intersections” command of the “Analyze Skeleton” plug-in.^25^

### 2.5 Infrared Spectroscopy

The chemical structures of bacterial cellulose samples obtained from the control culture, MonoCell, and GradCell, were verified using infrared (IR) spectroscopy, performed on a Thermo Fisher Scientific Nicolet 6700 spectrometer. IR spectra were collected over a wavelength range of 500–4000 cm^–1^, using both absorption and transmission modes to enhance data quality. Each spectrum consisted of 64 scans at a resolution of 4 cm^–1^, ensuring detailed spectral data. Post-acquisition, the spectra underwent several corrections to improve accuracy and interpretability. Attenuated total reflectance (ATR) correction was applied to adjust for the penetration depth of the IR beam into the sample. Smoothing techniques were employed to reduce noise, and baseline corrections were made to normalize the spectra. These preprocessing steps are crucial for accurate peak identification and quantification, providing a reliable analysis of the chemical structure of bacterial cellulose.^26^

### 2.6 Statistical Analysis

Data are presented as mean values and standard deviation. Statistical significance between sample populations is evaluated by using the online ANOVA test. The normality of data is checked by the Shapiro–Wilk test (*p*-values <0.05 indicate non-normal distribution). Differences are considered statistically significant for *p*-values <0.05. All the experiments are conducted in triplicate. The Pearson correlation coefficient between the ultrastructural and optical parameters is measured in Origin.

## 3 Results

### 3.1 Microfluidic Device Validation for Bacterial Cellulose Production

The design of the MonoCell device ensured efficient nutrient flow and oxygen exchange, creating a suitable environment for the culture of *K. xylinus*. The device featured a double-layer of PDMS structure and a central microchannel, including an observation chamber to monitor the cellulose synthesis process. A PC membrane separated the upper and lower layers, preventing the bacterial suspension from being carried away by the flow. Figure 1A shows a schematic illustration of the microfluidic device.

**Figure 1:**
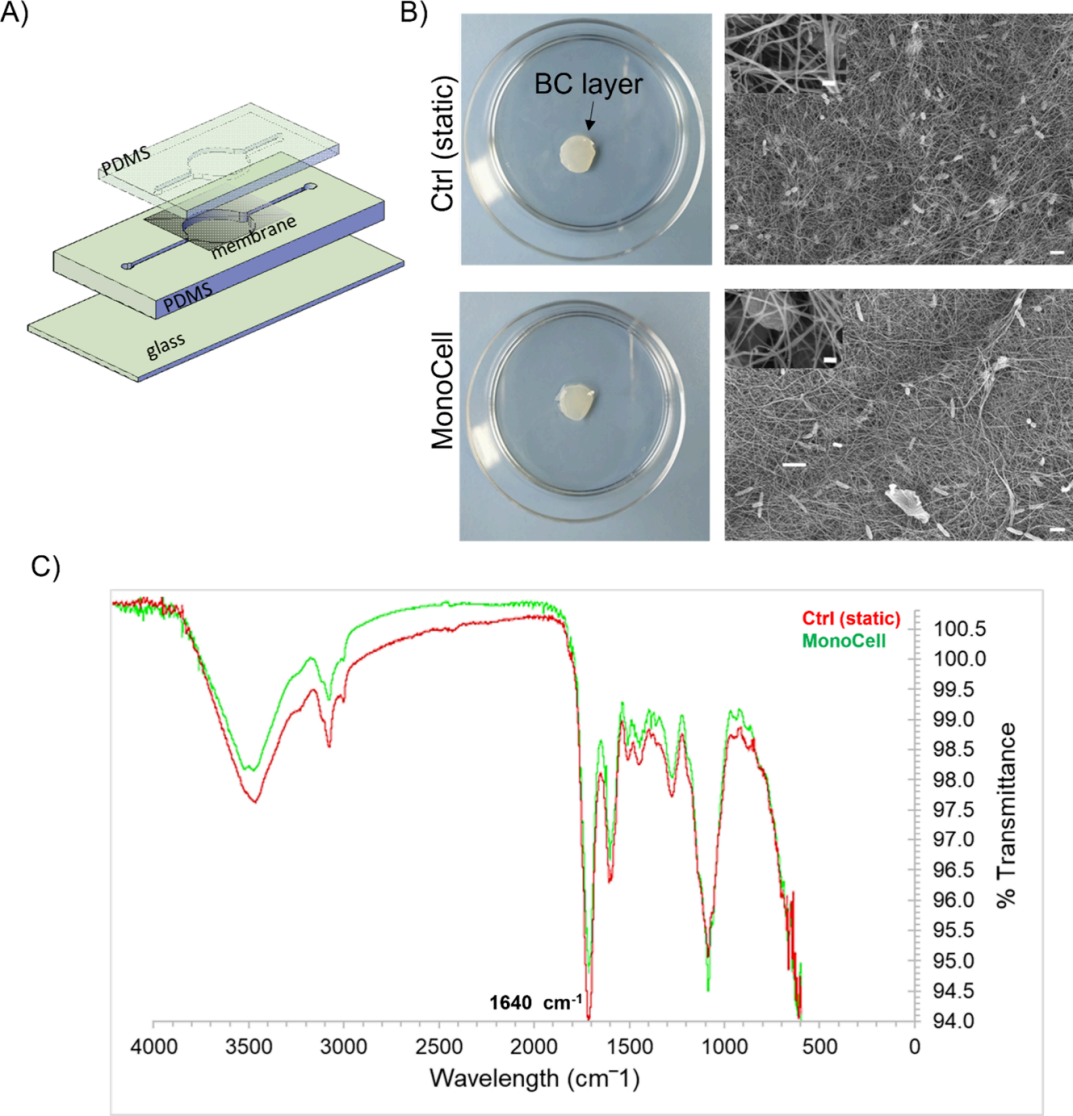
(A) Schematic illustration of the double layer PDMS single chip. (B) SEM images of both control (static) and microfluidic-chip (MonoCell) derived cellulose samples; scale bar: 200 nm. (C) IR spectra of both control and microfluidic-chip derived cellulose samples. All images were generated by the authors (BC, FM, EL).

To validate the effectiveness of the MC device for cellulose production, a comparative analysis was performed between the control culture and the chip-based culture. The control culture was established using a traditional static culture method by culturing *K. xylinus* in a multiwell plate containing HS medium. For chip-based culture, MonoCell was connected to a peristaltic pump, ensuring consistent flow rates and nutrient delivery, which were crucial for maintaining the culture’s viability and productivity over the 72 h period. The culture conditions were maintained at 30 °C in a humidified incubator (>80% humidity) for 72 h.

SEM images provided a detailed comparison of the cellulose structure produced in both the control and chip-based cultures (Figure 1B). The chip-based culture (bottom images) generated an organized and dense cellulose network comparable to the control culture (top images), indicating that the microfluidic device did not perturb cellulose matrix density and fiber organization.

The chemical structure of the bacterial cellulose produced in both cultures was verified by using infrared (IR) spectroscopy. The IR spectra (Figure 1C) were collected over a wavelength range of 500–4000 cm^–1^. Both spectra showed a characteristic peak at 1640 cm^–1^, confirming the presence of cellulose. The spectral data from the chip-based culture (green line) closely matched those of the control culture (red line), demonstrating that the chemical composition of the cellulose is maintained within the microfluidic device. These results validate the MonoCell as a reliable system for the culture of *K. xylinus*, providing a controlled environment for cellulose production and organization.

### 3.2 Online Monitoring of Cellulose Synthesis

MonoCell device enabled real-time monitoring of cellulose fibril synthesis through confocal microscopy, providing valuable insights into the dynamics of bacterial cellulose production. This monitoring is facilitated by the optically accessible structure of the device, allowing for detailed observation and analysis. Calcofluor white staining was employed to visualize the cellulose fibrils synthesized by *K. xylinus* during growth in MonoCell.

Figure 2A represents the schematic representation of the loading process of the *K. xylinus* suspension supplemented with Calcofluor white (0.02%). In the first step, the mixed suspension (*K. xylinus* + Calcofluor white dye) was added to the lower layer by pipetting to fill the observation chamber. In the second step, small polymeric caps were inserted into the inlet and outlet to seal the chip. Finally, the upper layer was connected to a peristaltic pump, and the whole setup was placed inside the incubator stage of the confocal microscope to start the monitoring process.

**Figure 2:**
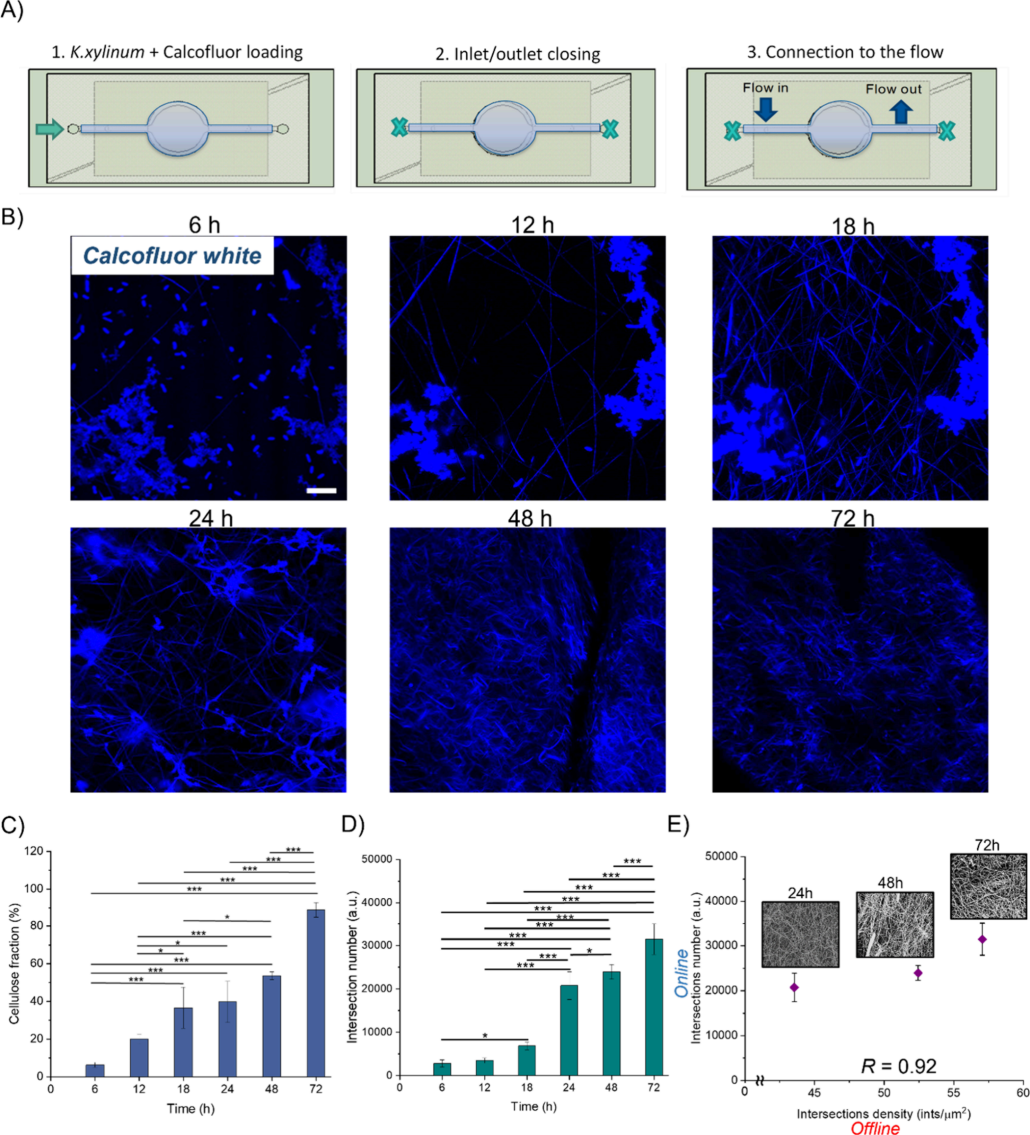
(A) Loading process of the *K. xylinus* + Calcofluor white dye in the MonoCell for the online monitoring of cellulose synthesis. (B) Confocal images of the synthesized fibrils captured at 6, 12, 18, 24, and 72 h, scale bar 10 μm. Quantification of cellulose fraction (C) and fiber intersection number (D) over time from confocal analysis, (D) Fiber intersection number obtained by confocal images analysis vs intersection density values obtained from SEM analysis at 24, 48, and 72 h. (E) R is the Poisson correlation coefficient between the online and offline parameters. Results were presented as mean values and standard deviation. Statistical significance is assessed through the ANOVA test (***p < 0.005, **p < 0.01, *p < 0.05, not significant when not shown, *n* ≥ 3 for condition). All images were generated by the authors (BC, FM).

Confocal microscopy images, captured at specific time points (6, 12, 18, 24, 48, and 72 h), revealed the progressive formation and organization of cellulose fibrils over time (Figure 2B). Image analysis of quantitative cellulose fraction percentages obtained by confocal images confirmed both the continuous production of cellulose and its accumulation, highlighting the dynamic biofilm maturation process in the microfluidic device (Figure 2C).

After 6 h, initial fiber formation was observed, appearing rare, accounting for approximately 8% of the cellulose fraction. By 12 h, the fibers became denser, forming a network, with the cellulose fraction rising to 20%. At 18–24 h, the fibril network became denser and more interconnected, with the cellulose fraction stabilizing around 40%. After 48–72 h, a highly dense and interconnected fiber matrix network with the cellulose fraction reaching 90%.

Additionally, to further validate the feasibility of MonoCell, the fiber intersection number values were calculated from the same confocal images. The results revealed an increasing trend over time (Figure 2D), according to a rise in cellulose production (Figure 2C). These data were then correlated with offline SEM analysis of cellulose layers collected at 24, 48, and 72 h. These time points were chosen to ensure a well-formed, structurally stable, and easily manageable cellulose layers. The correlation graph (Figure 2E) showed a strong positive correlation (*R* = 0.92), confirming the reliability of the *online* method for detecting structural information on cellulose networks, without the need for additional *offline* procedures.

### 3.3 Multicondition Gradient Mixing Chip: Effect of Yeast Extract and Glucose Concentration on the Cellulose Matrix

The GradCell device used in this work was designed with AutoCAD software, featuring an upstream concentration gradient generator and a downstream cell culture module (Figure 3). GradCell was designed to generate a concentration gradient across five culture chambers, enabling the stimulation of *K. xylinus* growth cultures under different conditions. This setup allowed us to investigate the effects of different concentrations of either yeast extract or glucose on the bacterial culture and cellulose matrix production.

**Figure 3:**
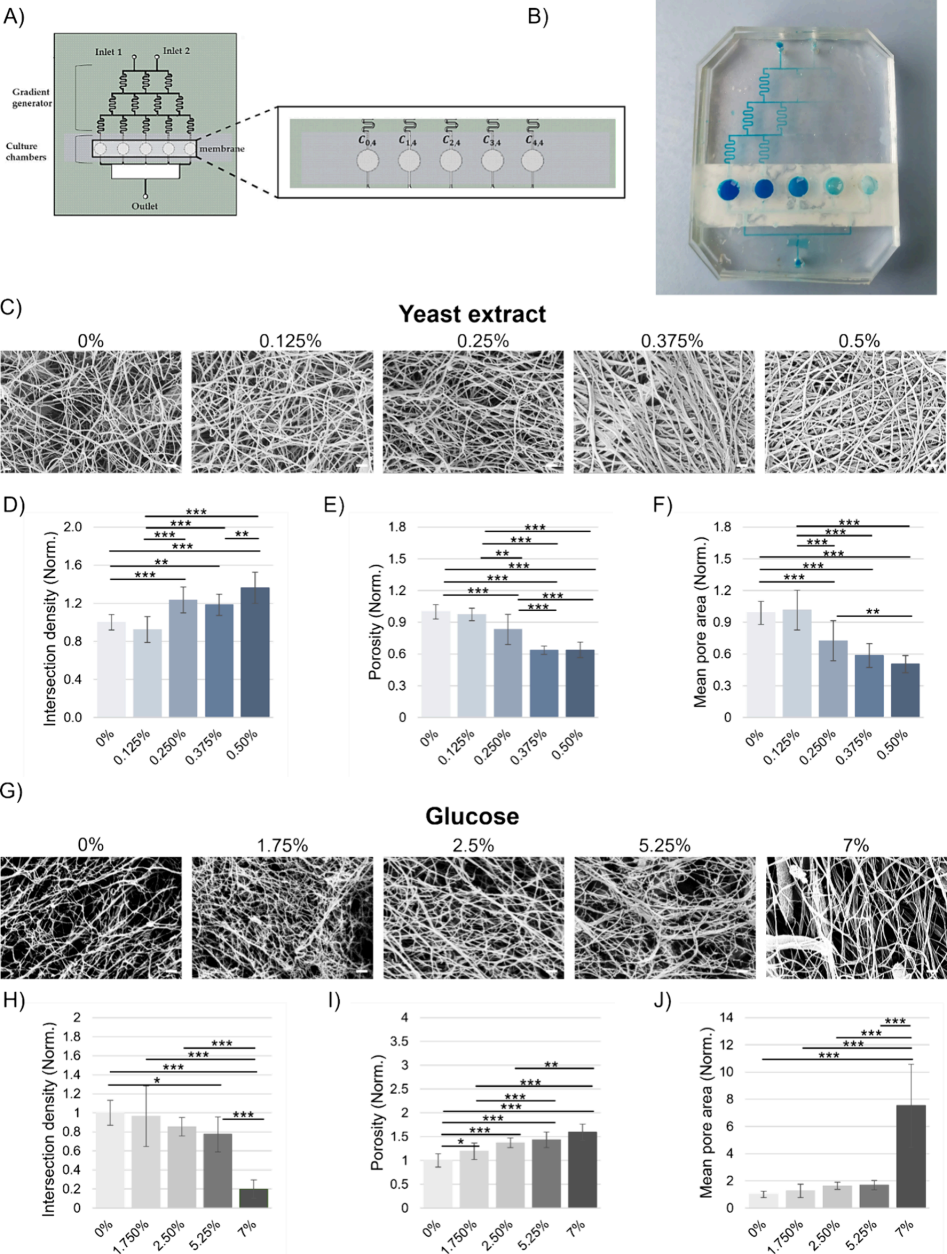
(A) Schematic illustration of GradCell, designed to simultaneously test different growth culture conditions across five separate chambers; (B) photograph of the chip filled with blue dye, visualizing the flow through the chambers. (C) SEM images of the cellulose samples produced in each of the five culture chambers under varying yeast extract concentrations (from 0 to 0.5%). Scale bar: 200 nm, (D) quantitative analysis of the intersection density of the cellulose fibers within each sample, showing changes across different yeast extract concentrations, (E) quantitative analysis of the porosity of cellulose samples cultured in each of the five chambers, highlighting the impact of yeast extract concentration on structural porosity. (F) Mean pore area measurements for the cellulose samples from each chamber, demonstrating how yeast extract concentration influences pore size. (G) SEM images of cellulose matrices produced under varying glucose concentrations (0, 1.75, 2.5, 5.25, and 7%). Scale bar: 200 nm. (H) Quantitative analysis of intersection density across different glucose concentrations. (I) Porosity analysis of the cellulose matrices with different glucose concentrations. (J) Mean pore area measurements across different glucose concentrations. Results were presented as mean values and standard deviation. Statistical significance is assessed through the ANOVA test (***p < 0.005, **p < 0.01, *p < 0.05, not significant when not shown, *n* ≥ 3 for condition). All images were generated by the authors (BC, FM).

Figure 3A depicts the schematic illustration of the design of the device, which included two inlets connected to a gradient generator, leading to a network of serpentine channels that delivered solution with varying concentrations into the culture chambers (*C*_0,4_, *C*_1,4_, *C*_2,4_, *C*_3,4_, and *C*_4,4_). The picture on the right (Figure 3B) displayed the device, highlighting the distribution of dyed solutions to visualize the concentration gradient across the chambers.

Figure 3C reports SEM images of the cellulose matrices produced in the presence of increasing concentrations of yeast extract, ranging from 0 to 0.5% (w/v). As the yeast extract concentration increased, notable changes in the cellulose matrix structure were observed.

Quantitative analysis allowed for the measurement of ultrastructural parameters such as intersection density, porosity, and mean pore area. Intersection density was referred to as the number of fiber intersections or cross-links per unit area and provided a quantitative measure of how densely the fibers in the matrix are interconnected or overlapped. Our results indicated that in the absence of yeast extract, the matrices generated appear less dense, with fewer fiber intersections, as confirmed by the intersection density graph (Figure 3D), which shows a similar intersection density for both samples at 0 and 0.125% concentration of yeast extract. As the yeast extract concentration increased to 0.25%, the density of fiber’s intersections increased by 1.2-fold, indicating a more tightly packed matrix. At the highest concentrations of yeast extract (0.375–0.5%), the intersection density increased by 1.4-fold, reflecting a highly interconnected and dense fiber network.

Porosity refers to the proportion of a material’s volume that is occupied by voids or empty spaces, rather than solid material. In this study, porosity is used to assess how the concentration of yeast extract or glucose affects the openness or compactness of the cellulose matrix. As observed in the SEM images and supported by quantitative analysis, changes in the porosity were correlated with the concentrations of these supplements. In detail, as the concentration of yeast extract increased, porosity tended to decrease, indicating a more compact matrix with fewer and smaller pores. Conversely, increasing glucose concentrations led to higher porosity, suggesting a more open structure with larger voids. As reported in the porosity graph (Figure 3E), the cellulose matrix at 0% yeast extract exhibited the highest porosity, suggesting that in the absence of yeast extract, the matrix formed with larger and more numerous voids, resulting in a more open and less dense structure. As the concentration of yeast extract increased, a clear and consistent decrease in porosity was observed. The results of the GradCell experiment conducted with varying glucose concentrations (0, 1.75, 3.5, 5.25, and 7%) revealed significant effects on the structural properties of the cellulose matrix produced by *K. xylinus* as reported in Figure 3G.

The intersection density (Figure 3H) decreased slightly as the glucose concentration rose from 0 to 7%. This indicated that at lower glucose concentrations, there was a slight reduction in network density, leading to fewer fiber intersections. This effect became more pronounced at 5.25% of glucose. At 7% glucose, the intersection density was the lowest among all concentrations. These findings suggested that higher glucose levels resulted in a less interconnected cellulose matrix. Additionally, the porosity (Figure 3I) of the cellulose matrix increased significantly as the glucose concentration rises. At 7% glucose, the porosity was at its highest. This indicated that higher glucose concentrations led to the formation of a more open matrix with larger voids between the fibers.

Similarly, the mean pore area (Figure 3J) showed a marked and significant increase as glucose concentrations rise, reaching its peak at 7%. This indicated that the pores within the cellulose matrix became larger as the glucose availability increases. The larger pore sizes at higher glucose levels further supported the observation of an increased porosity and decreased intersection density.

## 4 Discussion

In this study, we developed and validated a scalable microfluidic platform designed to generate bacterial cellulose layers (BC) under controlled conditions and to characterize the resulting cellulose matrix across a range of nutrient concentrations. Although microfluidic systems have been employed to cultivate bacterial cells^27^ and produce bacterial cellulose under shear stress conditions,^22^ their applications remain limited. Moreover, unlike microfluidic setups that rely on physical constraints such as pillars to direct biofilm growth, our system eliminates the need for these structures, allowing for the formation of a continuous bacterial cellulose network. Current approaches focus primarily on detecting bacterial presence,^28,29^ studying the effects of antimicrobial drugs,^30^ and producing BC microspheres.^31^ However, these systems lack the capability for real-time monitoring and control of BC production, limiting their potential for advanced applications. Our microfluidic system enables a more comprehensive optimization of BC characteristics, providing real-time monitoring and high-throughput analysis, where multiple variables can be tested simultaneously, thereby accelerating the identification of optimal culture settings and enabling a more comprehensive optimization of BC characteristics for several applications.

Furthermore, this platform offers the advantage of scaling down the cellulose fermentation process, providing enhanced control of the bacteria growth culture parameters, which in turn allows for a more efficient and cost-effective cellulose fermentation process. Our findings highlight the versatility of the newly designed microfluidic system in optimizing the culture conditions for *K. xylinus* and manipulating key structural properties of the resulting cellulose. To explore this, we characterized the BC layers produced under dynamic culture conditions by using a combination of scanning electron microscopy (SEM) and confocal laser scanning microscopy. SEM, a well-established technique for the structural characterization of bacterial cellulose,^32^ provides high-resolution imaging of surface morphology.^33^ However, it typically requires sample removal from the culture environment and extensive preparation, which may introduce artifacts and limit the ability to capture dynamic structural changes.^34^ In contrast, the optical accessibility of our cellulose-based microfluidic chip allows for real-time detection of structural information, enabling a more accurate assessment of BC formation and evolution under physiologically relevant conditions.^35,36^

By incorporating Calcofluor White staining, a cellulose-specific fluorescent dye, we were able to monitor the real-time increase in cellulose production and fiber densification in *K. xylinus* cultures by using a confocal microscope. Furthermore, the correlation observed between optical and electron microscopy data confirms the ability of the microfluidic system to provide insight into the microstructure of bacterial cellulose layers without altering or disrupting the sample’s native environment. Similarly, our recent study found a correlation between SHG and SEM analyses of Symbiotic Culture of Bacteria and Yeast (SCOBY) bioflocs, enabling the assessment of their structural characteristics without altering the sample.^37^ To further explore the impact of growth culture conditions on BC characteristics, we enhanced the microfluidic platform by creating a gradient mixing chip with multiple chambers. This enabled us to test the effects of different concentrations of two key components of the culturing medium—yeast extract and glucose—which are known to impact cellulose yield.^38^ The BC layers obtained from the GradCell were then structurally characterized by SEM analyses to validate the efficiency of the device. Yeast extract serves as a rich source of nitrogen and other growth factors, enhancing the metabolic activity of *K. xylinus* and promoting cellulose biosynthesis.^39,40^ Our results indicate that increasing yeast extract concentrations leads to the formation of a more compact cellulose network, characterized by smaller and fewer pores and a higher fiber intersection density. The mean pore area represents the average size of voids within a material’s structure and is a key parameter for understanding permeability, diffusion properties, and mechanical strength. Larger pores may enhance the permeability but reduce the structural integrity, whereas smaller pores contribute to a denser and potentially stronger matrix. Similar to the porosity, higher yeast extract concentrations result in a reduction of the mean pore area, leading to smaller and more compact pores. This suggests that increasing yeast extract levels promotes the formation of a denser cellulose network with reduced porosity.

These results align with previous studies, which suggest that higher yeast extract concentrations promote cellulose production.^41^ Furthermore, the GradCell experiment with different glucose concentrations revealed that higher glucose levels significantly affect the structural properties of the cellulose matrix. Higher glucose levels lead to a decrease in intersection density, an increase in porosity, and a larger mean pore area, resulting in a more open and less dense matrix structure. These findings also align with previous studies, which reported that higher initial glucose concentrations inhibit cellulose production.^42–45^ This effect is attributed to the conversion of glucose to gluconic acid, whose accumulation alters the pH of the fermentation medium, ultimately reducing cellulose production.

The dynamic relationship between the glucose concentration and the structural properties of the BC matrix underscores the pivotal role that glucose plays in controlling cellulose morphology. At higher glucose concentrations, the cellulose matrix becomes more porous and less interconnected. This knowledge is valuable for applications where the porosity and permeability of the cellulose matrix are critical, such as in filtration systems, tissue engineering scaffolds, or other biotechnological applications where specific structural properties are desired.^46^

In tissue engineering, a cellulose matrix with controlled porosity and pore size is crucial, as it influences cell attachment, rapid nutrient diffusion for cell survival, growth, and cell migration.^47,48^ Higher porosity, achievable through glucose concentration modulation in this platform, creates an open matrix structure with larger pores, promoting nutrient diffusion and cellular ingrowth—a critical feature for scaffolds intended to support rapid tissue formation. Conversely, applications requiring barrier functions, such as controlled drug release, would benefit from a denser, less porous cellulose structure, easily attainable by modulating yeast extract levels to produce a tighter fiber network.^49^ Our data suggest that the structural adaptations of the BC matrix are driven by the interplay between nutrient availability and controlled conditions within the microfluidic system. MonoCell effectively facilitates the manipulation of bacteria growth culture conditions to induce specific structural outcomes, providing a powerful tool for optimizing bacterial cellulose production for various biotechnological applications. Beyond the experimental results presented here, our real-time monitoring approach could also provide the basis for kinetic modeling of *K. xylinus* growth and cellulose biosynthesis. Classical Monod-type models, which describe microbial growth as a function of substrate availability, have already been applied to bacterial cellulose production and could be adapted to integrate our microfluidic measurements of glucose consumption and biomass accumulation.^50^ Coupling such models with Luedeking–Piret equations would allow the prediction of cellulose productivity as a function of culture conditions, while empirical approaches such as Gompertz-type models have also shown high accuracy in describing pellicle formation.^51^ In addition, computational fluid dynamics simulations of our device could help capture nutrient gradients and flow-related effects, providing critical parameters for model calibration.

From an economic and scalability perspective, our platform is intended as a high-throughput optimization tool to guide large-scale processes. Based on our experiments, the MonoCell device yielded bacterial cellulose layers with an estimated productivity of ∼28.3 mg/day, calculated by considering the reported density of bacterial cellulose (1.25 g/cm^3^)^52^ and the volume of the central chamber of our device (0.067 cm^3^), which was fully filled by the cellulose layer after 72 h of culture. This value corresponds to the same order of magnitude of volumetric productivity typically reported for traditional batch cultures of *K. xylinus*, confirming that the microfluidic platform does not alter the kinetics of BC synthesis. Additionally, the maximum operating flow rate of the current chip is estimated to be in the range of 10–200 μL/min, beyond which shear stress may impair cell adhesion and cellulose network formation. From a cost perspective, prototyping microfluidic devices in PDMS or glass costs between 1 and 5 dollars per chip, whereas thermoplastic (e.g., PMMA) molding demands higher upfront costs (10–100 dollars per mold) but is economically efficient at scale with costs <1 dollar per chip.^53,54^ In addition to the low fabrication cost of the presented microfluidic platform, the reduced reagent consumption and the potential for parallelization offer significant industrial value by accelerating the identification of optimal culture conditions before scale-up in conventional bioreactors.

By offering a scalable, precise, and high-throughput approach, the GradCell allows for the simultaneous testing of multiple variables and offers valuable awareness of the dynamic and progressive nature of cellulose biosynthesis. This platform holds significant potential for advancing research in bacterial cellulose applications, ranging from materials science to medical and industrial uses.

## 5 Conclusions

This study demonstrates the use of a scalable MonoCell platform designed for the controlled production and characterization of bacterial cellulose synthesized by *K. xylinus*. The microfluidic approach provides significant advantages over traditional bioreactor-based methods, allowing for precise control of environmental conditions, real-time monitoring, and a substantial reduction in resource consumption. Structural analyses via confocal and SEM imaging reveal that varying concentrations of yeast extract and glucose can distinctly modulate the density, porosity, and overall architecture of the BC matrix. Specifically, higher yeast extract concentrations produce a denser, more compact structure, while increased glucose levels create a more porous and open matrix. MonoCell provides a resource-efficient, versatile solution for BC production, supporting high-throughput testing and fine-tuning of culture conditions that cater to specific application needs in biomedical and cosmetic fields. By enabling the modulation of cellulose matrix properties, this approach supports targeted applications, including tissue engineering, drug delivery, and other biotechnological uses for which structural customization is essential. Overall, this work establishes a novel and adaptable tool for bacterial cellulose production, expanding its potential for both research and industrial applications.
